# Alterations in *EGFR* and *PDGFRA* are associated with the localization of contrast-enhancing lesions in glioblastoma

**DOI:** 10.1093/noajnl/vdad110

**Published:** 2023-09-02

**Authors:** Ryutaro Makino, Nayuta Higa, Toshiaki Akahane, Hajime Yonezawa, Hiroyuki Uchida, Tomoko Takajo, Shingo Fujio, Mari Kirishima, Taiji Hamada, Hitoshi Yamahata, Kiyohisa Kamimura, Takashi Yoshiura, Koji Yoshimoto, Akihide Tanimoto, Ryosuke Hanaya

**Affiliations:** Department of Neurosurgery, Graduate School of Medical and Dental Sciences, Kagoshima University, Kagoshima, Japan; Department of Neurosurgery, Graduate School of Medical and Dental Sciences, Kagoshima University, Kagoshima, Japan; Department of Pathology, Graduate School of Medical and Dental Sciences, Kagoshima University, Kagoshima, Japan; Center for Human Genome and Gene Analysis, Kagoshima University Hospital, Kagoshima, Japan; Department of Neurosurgery, Graduate School of Medical and Dental Sciences, Kagoshima University, Kagoshima, Japan; Department of Neurosurgery, Graduate School of Medical and Dental Sciences, Kagoshima University, Kagoshima, Japan; Department of Neurosurgery, Graduate School of Medical and Dental Sciences, Kagoshima University, Kagoshima, Japan; Department of Pathology, Graduate School of Medical and Dental Sciences, Kagoshima University, Kagoshima, Japan; Department of Pathology, Graduate School of Medical and Dental Sciences, Kagoshima University, Kagoshima, Japan; Department of Neurosurgery, Graduate School of Medical and Dental Sciences, Kagoshima University, Kagoshima, Japan; Department of Advanced Radiological Imaging, Graduate School of Medical and Dental Sciences, Kagoshima University, Kagoshima, Japan; Department of Advanced Radiological Imaging, Graduate School of Medical and Dental Sciences, Kagoshima University, Kagoshima, Japan; Department of Radiology, Graduate School of Medical and Dental Sciences, Kagoshima University, Kagoshima, Japan; Department of Neurosurgery, Graduate School of Medical Sciences, Kyushu University, Fukuoka, Japan; Department of Pathology, Graduate School of Medical and Dental Sciences, Kagoshima University, Kagoshima, Japan; Center for Human Genome and Gene Analysis, Kagoshima University Hospital, Kagoshima, Japan; Department of Neurosurgery, Graduate School of Medical and Dental Sciences, Kagoshima University, Kagoshima, Japan

**Keywords:** glioblastoma, multicentric glioma, multifocal glioma, neural stem cell, subventricular zone

## Abstract

**Background:**

Glioblastoma (GBM) is a malignant brain tumor, with radiological and genetic heterogeneity. We examined the association between radiological characteristics and driver gene alterations.

**Methods:**

We analyzed the driver genes of 124 patients with *IDH* wild-type GBM with contrast enhancement using magnetic resonance imaging. We used a next-generation sequencing panel to identify mutations in driver genes and matched them with radiological information. Contrast-enhancing lesion localization of GBMs was classified into 4 groups based on their relationship with the subventricular zone (SVZ) and cortex (Ctx).

**Results:**

The cohort included 69 men (55.6%) and 55 women (44.4%) with a mean age of 66.4 ± 13.3 years. *EGFR* and *PDGFRA* alterations were detected in 28.2% and 22.6% of the patients, respectively. Contrast-enhancing lesion touching both the SVZ and Ctx was excluded because it was difficult to determine whether it originated from the SVZ or Ctx. Contrast-enhancing lesions touching the SVZ but not the Ctx had significantly worse overall survival than non-SVZ lesions (441 days vs. 897 days, *P* = .002). GBM touching only the Ctx had a better prognosis (901 days vs. 473 days, *P* < .001) than non-Ctx lesions and was associated with *EGFR* alteration (39.4% vs. 13.2%, *P* = .015). Multiple contrast lesions were predominant in *PDGFRA* alteration and *RB1*-wild type (*P* = .036 and *P* = .031, respectively).

**Conclusions:**

*EGFR* alteration was associated with cortical lesions. And *PDGFRA* alteration correlated with multiple lesions. Our results suggest that clarifying the association between driver genes and tumor localization may be useful in clinical practice, including prognosis prediction.

**Key Points**

- Cortical lesions in glioblastoma were associated with *EGFR* alteration and *PTEN* loss.
- Multiple contrast-enhancing lesions were associated with *PDGFRA* alteration.
- Alterations in *EGFR* and *PDGFRA* may affect imaging findings and patient prognosis.

**Importance of the Study**

Glioblastoma (GBM) is a malignant brain tumor with genetic heterogeneity. Receptor tyrosine kinases have been implicated in the pathogenesis of glioblastoma, and *EGFR* amplification has been added to the diagnostic criteria of the 2021 World Health Organization classification of tumors of the central nervous system. GBMs have radiologically heterogeneous features; however, their relationship with molecular genetics remains unclear. We analyzed *IDH* wild-type glioblastomas and contrast-enhancing lesions shown using magnetic resonance imaging and found that cortical lesions showed significantly higher *EGFR* alteration and *PTEN* loss, and periventricular lesions showed higher *CDK4* amplification. In addition, multiple lesions were more common in *PDGFRA* alteration and *RB1* wild type. The alterations in *EGFR* and *PDGFRA* expression showed contrasting characteristics regarding Ki-67 expression and resection rates. Our report is the first radiogenomic study to show that receptor tyrosine kinases are potentially involved in the localization, multiplicity, and proliferative capacity of GBMs.

Glioblastoma (GBM) is a primary brain tumor with a poor prognosis and heterogeneous clinical and pathological characteristics. Genetic analyses have improved our understanding of the molecular heterogeneity of GBM. In addition, GBM has a heterogeneous appearance on radiological examination, and the relationship between these findings and the genetic background remains unclear. Changes in receptor tyrosine kinases (RTKs) such as *EGFR*, *PDGFR*, *MET*, and *FGFR* occur in approximately 90% of GBMs. These alterations, particularly the more common ones in *EGFR* and *PDGFRA*, serve as valuable diagnostic markers.^1,2^ The diagnosis of GBM has shifted from histological evaluation to molecular genetics evaluation. Several studies have shown the importance of *EGFR*.^3,4^ In the 2021 World Health Organization (WHO) Classification of Tumors of the Central Nervous System, *EGFR* amplification has been added to the diagnostic criteria of *IDH* wild-type GBM.^5^

The localization of GBM can be classified according to whether it is in contact with the subventricular zone (SVZ) or not, and the former tends to recur as multiple lesions^6^ with a short survival time.^7–10^ Neural stem cells (NSCs) in the SVZ have genetically distinct characteristics from other localizations and may influence the development and progression of GBM.^11,12^

Multiple lesions, including multifocal and multicentric GBM, are found in 0.5%–35% of all GBMs^13–17^ and are associated with a worse prognosis than solitary lesions.^14^ Several major genetic variants, including *PTEN* loss, *TERT* mutation,^18^ and *EGFR* mutation,^19^ are found in multiple lesions in GBMs. However, the relationship between these genetic changes and the localization or multiplicity of contrast-enhancing lesions (CELs) remains unclear.

The combination of radiological findings with genetic information is a recent topic, and research is advancing with the widespread use of next-generation sequencing (NGS) panels. We aimed to determine the association between genetic information and the multiplicity, localization, and extent of GBM invasion using NGS panels.

## Materials and Methods

This retrospective study was approved by the Institutional Review Board of Kagoshima University Hospital (approval no. 180104) and complied with the guiding principles of the Declaration of Helsinki. Furthermore, written informed consent was obtained from each patient. We collected 124 samples diagnosed with supratentorial, *IDH* wild type, and WHO grade 4 GBM from the central nervous system tumor tissue bank at Kagoshima University Hospital. The tumors were fixed with phosphate-buffered 10% formalin for 24 h and processed for paraffin embedding, followed by sectioning for hematoxylin and eosin staining. All tissues were histologically evaluated by board-certified pathologists to ensure an estimated tumor cell content of ≥30%.

### Next-Generation Sequencing

NGS was performed using an amplicon-based glioma-tailored 48-gene (version 2) or 50-gene panel (version 3) (QIAGEN) with 2244 primers for the regions of interest (161179 bp) and an average exon coverage of 99.95%.^20,21^ The amplicon sequences were aligned to the human reference genome GRCh37 (hg19) in the target region of the sequence. Data were analyzed using the QIAGEN Web Portal service (https://www.qiagen.com/ja-us) and Mitsubishi Space Software (Amagasakihttps://www.mesw.co.jp).

### Methylation-Specific Polymerase Chain Reaction

Bisulfite conversion of the extracted genomic DNA was performed using the EpiTect Bisulfite Kit (Qiagen). The converted genomic DNA was amplified for the target O6-methylguanine-DNA methyltransferase promoter (*MGMT*p) region with primers specific for the methylated or unmethylated template using KOD One® PCR Master Mix (Toyobo). Two pairs of primers specific for methylated or unmethylated MGMTp regions were used for methylation-specific polymerase as previously reported.^22^ The amplification was performed with an initial denaturation at 98°C for 1 min, followed by 40 cycles of 98°C for 10 s and 64°C for 5 s. MCE-202 MultiNA (Shimadzu) was used for the analysis.

### Radiological Analysis

Based on previous reports, CELs of GBM were classified into 4 groups: group I, the CEL extends from the atrium SVZ to the pia; group II, the CEL touches the SVZ but does not involve the cortex (Ctx); group III, the CEL invades the Ctx and reaches the pia but does not touch the SVZ; and group IV, the CEL does not touch either the Ctx or SVZ (Supplementary Figure 1).^6,8^ We first classified the entire cohort of 124 patients into the 4 categories.

GBM can be classified into 3 categories based on their multiplicity: solitary, multifocal, and multicentric. Multifocal GBM is defined as a CEL that appears remote to some extent but is microscopically connected and contained within a common hyperintense area on a T2-weighted image (T2WI). Multicentric GBM refers to CELs in different lobes or bilateral brains that do not have potential contacts, such that they have a common high-intensity area on a T2WI.^15,17,23^ All image evaluations were performed by 2 board-certified neurosurgeons (N.H. and H.Y.) using a PACS system (Synapse PACS 4.1.3; Fuji Film Medical Systems). The interval between imaging and tissue collection was within 1 month in all cases.

### Data Analysis

Statistical analyses were performed using the GraphPad Prism9 software (MDF Co., Ltd.). The relationship among location, multiplicity, and genetic information was analyzed using χ^2^ tests and *t*-test. The log-rank test was used to analyze overall survival (OS). Differences with *P* < .05 were considered statistically significant.

## Results

### Genetic Information

Gene alterations were identified in 124 samples as follows: 35 displayed *EGFR* alterations (25 amplifications, 5 mutations, 5 both), 28 had *PDGFRA* alterations (13 amplifications, 6 mutations, 9 both), and 83 exhibited *TERT*p mutations. Additionally, 62 samples showed *PTEN* loss (31 losses of heterozygosity, 31 homozygous deletions). *RB1* alterations were present in 52 samples (24 losses of heterozygosity, 14 homozygous deletions, 7 mutations, 4 losses of heterozygosity with mutations, 3 homozygous deletions with mutations), while 60 samples had *TP53* alterations (3 losses of heterozygosity, 1 homozygous deletion, 53 mutations, 3 losses of heterozygosity with mutations). There were 23 samples with *CDK4* amplifications and 3 with *CDK6* amplifications. *CDKN2A/2B* homozygous deletions were found in 57 samples, and *MGMT*p methylation was observed in 84 samples.

### Clinical and Genetic Characteristics of GBM

**Figure 1.**
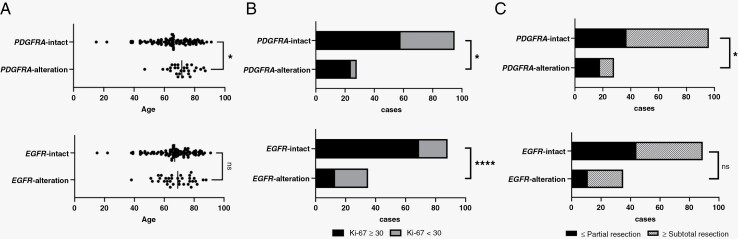
Clinical features of GBM depending on *EGFR* and *PDGFRA* status. (A) Patients with *PDGFRA* alteration were significantly older than those with intact *PDGFRA* (*P* = .020, unpaired *t*-test). *EGFR* alteration did not show a significant difference in age compared with that of *EGFR* intact. (B) Ki-67 labeling index was classified into higher (≥30) and lower (<30) groups. *EGFR* and *PDGFRA* alterations showed lower and higher Ki-67 labeling index scores, respectively (*P* < .0001, *P* = .021: Fisher’s exact test). (C) We classified the patients into 2 groups: subtotal or greater resection and partial or less resection. *PDGFRA* alteration had a significantly poorer resection rate (*P* = .019). *EGFR* alteration had better removal rates; however, the difference was not statistically significant (*P* = .075).

Overall, 124 patients were included in this study (Table 1). The mean age of the patients was 66.4 ± 13.3 years. There were 69 men (55.6%) and 55 women (44.4%). The number of patients in each group based on the localization of CELs was as follows: group I, *n* = 53 (43%); group II, *n* = 33 (27%); group III, *n* = 33 (27%); group IV, *n* = 5 (4%). The preoperative Karnofsky performance score (KPS) information was available in 124 patients, and 55 (44.4%) patients had a good KPS (≥80) preoperatively. The removal rates were divided into biopsy (*n* = 18, 14.5%), partial resection (*n* = 37, 29.8%), subtotal resection (*n* = 17, 13.7%), and total resection (*n* = 52, 41.9%). The Ki-67 labeling index information was available in 123 patients (range 6‒90%, mean 38.9 ± 18.2%). We defined a high level of Ki-67 as Ki-67 index ≥30. Higher Ki-67 levels were observed in older patients (*P* = .06), whereas the Ki-67 level was not associated with sex, localization, or OS. The driver gene alterations in the cohort are presented in Table 1. *EGFR* and *PDGFRA* alterations were detected in 28.2% and 22.6% of the patients, respectively. Patients with *PDGFRA* alteration were significantly older than those with intact *PDGFRA* (71.6 vs. 65.0, *P* = .020, unpaired *t*-test), whereas *EGFR* alteration was not significant (Figure 1A). *EGFR* alteration correlated with a lower Ki-67 index (*P* < .0001). In contrast, *PDGFRA* alteration resulted in higher Ki-67 expression (*P* = .021; Figure 1B). We classified the patients into 2 groups: subtotal or greater resection and partial or less resection. *PDGFRA* alterations were associated with significantly poorer resection rates (*P* = .019). *EGFR* alterations had better removal rates, although the difference was not statistically significant (*P* = .075; Figure 1C).

**Table 1.** Genetic and Radiographic Characteristics of the Study Cohort

		CEL group				Lesion type		
Variables	All	I	II	III	IV	Solitary	Multifocal	Multicentric
No. of patients	124	53	33	33	5	87	28	9
Mean age (y)	66.4 ± 13.3	67.3 ± 13.4	68.8 ± 11.6	63.2 ± 14.7	63.6 ± 7.4	65.9 ± 14.5	66.8 ± 9.1	70.9 ± 10.6
Sex (male/female)	69/55	29/24	22/11	15/18	3/2	48/39	16/12	5/4
Mean Ki-67 labeling index	38.9 ± 18.2	38.5 ± 17.2	40.4 ± 19.6	38.8 ± 18.0	36.6 ± 18.2	39.5 ± 18.3	37.0 ± 17.4	40.7 ± 18.3
Biopsy	18 (15%)	5 (9%)	10 (30%)	1 (3%)	2 (40%)	9 (10.3%)	4 (14.3%)	5 (55.6%)
Partial resection	37 (30%)	19 (36%)	13 (39%)	4 (12%)	1 (20%)	19 (21.8%)	14 (50.0%)	4 (44.4%)
Subtotal resection	17 (14%)	9 (17%)	3 (9%)	5 (15%)	0	12 (13.8%)	5 (17.9%)	0
Gross total resection	52 (42%)	20 (38%)	7 (21%)	23 (70%)	2 (40%)	47 (54.0%)	5 (17.9%)	0
*EGFR* alteration	35 (28.2%)	17 (32.1%)	4 (12.1%)	13 (39.4%)	1 (20.0%)	25 (28.7%)	8 (28.6%)	2 (22.2%)
*PDGFRA* alteration	28 (22.6%)	12 (22.6%)	9 (27.3%)	6 (18.2%)	1 (20.0%)	15 (17.2%)	9 (32.1%)	4 (44.4%)
*TERT*p mutation	83 (66.9%)	37 (69.8%)	21 (63.6%)	23 (69.7%)	2 (40.0%)	60 (69.0%)	19 (67.9%)	4 (44.4%)
*PTEN* loss	62 (50.0%)	27 (50.9%)	12 (36.4%)	22 (66.7%)	1 (20.0%)	46 (52.9%)	12 (42.9%)	4 (44.4%)
*RB1* alteration	52 (41.9%)	21 (39.6%)	15 (45.5%)	15 (45.5%)	1 (20.0%)	42 (48.3%)	9 (32.1%)	1 (11.1%)
*TP53* alteration	60 (48.4%)	23 (43.4%)	19 (57.6%)	17 (51.5%)	1 (20.0%)	43 (49.4%)	14 (50.0%)	3 (33.3%)
*CDK4* amplification	23 (18.5%)	7 (13.2%)	11 (33.3%)	5 (15.2%)	0	15 (17.2%)	6 (21.4%)	2 (22.2%)
*CDK6* amplification	3 (2.4%)	2 (3.8%)	0	1 (3.0%)	0	3 (3.4%)	0	0
*CDKN2A/2B* homozygous deletion	57 (46.0%)	26 (49.1%)	15 (45.5%)	14 (42.4%)	2 (40.0%)	42 (48.3%)	9 (32.1%)	6 (66.7%)
*MGMTp* methylation	84 (67.7%)	40 (75.5%)	21 (63.6%)	20 (60.6%)	3 (60.0%)	59 (67.8%)	19 (67.9%)	6 (66.7%)
Median OS (days)	618	716	441	901	566	688	488	540

Abbreviations: CEL, contrast-enhanced lesion; OS, overall survival.

### Differences in Clinical Background and Genetic Status Depending on Location Within the SVZ and Cortex

Comparisons between the SVZ (CEL Groups I and II) and non-SVZ groups (CEL Groups III and IV) are presented in Table 2. Patients with GBM in contact with the SVZ were older than those with GBM not in contact with the SVZ (*P* = .070, unpaired *t*-test). This group showed poor preoperative KPS (*P* < .001) and had difficulties achieving more than subtotal resection (*P* < .001). Moreover, there was no significant difference in the driver gene status between the SVZ and non-SVZ groups.

**Table 2.** Relationship Between Driver Gene Alterations and Localization of Contrast-Enhanced Lesions

Variables	All, *n* = 124	SVZ (Group I, II), *n* = 86	Non-SVZ (Group III, IV), *n* = 38	*P*-value
Mean age	66.4 ± 13.3	67.9 ± 12.7	63.2 ± 14.0	.073
Preoperative KPS ≥ 80	55 (44.4%)	29 (33.7%)	26 (68.4%)	<.001^***^
≥Subtotal resection	69 (55.6%)	39 (45.4%)	30 (79.0%)	<.001^***^
*EGFR* alteration	35 (28.2%)	21 (24.4%)	14 (36.8%)	.195
*PDGFRA* alteration	28 (22.6%)	21 (24.4%)	7 (18.4%)	.642
*RB1* alteration	52 (41.9%)	36 (41.9%)	16 (42.1%)	1.000
*PTEN* alteration	79 (63.7%)	53 (61.6%)	26 (68.4%)	.546
*CDK4* amplification	23 (18.5%)	18 (20.9%)	5 (13.2%)	.453
*CDK6* amplification	3 (2.4%)	2 (2.3%)	1 (2.6%)	1.000
*TERT*p mutation	83 (66.9%)	58 (67.4%)	25 (65.8%)	1.000
*TP53* alteration	60 (48.4%)	42 (48.8%)	18 (47.4%)	1.000
*MGMT*p methylation	84 (67.7)	61 (70.9%)	23 (60.5%)	.299
Median OS (days)	618	567	897	.037^*^

Abbreviations: KPS, Karnofsky performance score; OS, overall survival; SVZ, subventricular zone.

*P <.05, **P <.01, ***P <.001.

CEL group I touched the SVZ and Ctx, making it difficult to determine whether it originated from the SVZ or Ctx. Therefore, we excluded group I and re-examined the tumor localization and genetic characteristics. We compared group II, which was thought to have originated from the SVZ, with the non-SVZ groups (groups III and IV). Similarly, we compared group III, which was thought to have originated from the Ctx, with the non-Ctx groups (groups II and IV). Group II had fewer *EGFR* alterations (12.1% vs. 36.8%, *P* = 0.027) than the non-SVZ groups (groups III and IV; Table 3). *CDK4* amplification was more common in group II (*P* = .051) than in the non-SVZ groups; however, the difference was not statistically significant (Table 3). Group III showed significantly higher *EGFR* alteration (39.4% vs. 13.2%, *P* = .015) and *PTEN* loss (66.7% vs. 34.2%, *P* = .009) than the non-Ctx groups (Table 3). *PDGFRA*, *TP53*, *RB1*, *CDK4/6*, *CDKN2A/B*, and *TERT* promoters showed no significant differences in localization.

**Table 3.** Relationship Between Driver Gene Alterations and Localization of Contrast-Enhanced Lesions Excluding Group I

Variables	All, *n* = 71	Group II, *n* = 33	Non-SVZ (Group III, IV), *n* = 38	*P*-value
*EGFR* alteration	18 (25.4%)	4 (12.1%)	14 (36.8%)	.027^*^
*PDGFRA* alteration	16 (22.5%)	9 (27.2%)	7 (18.4%)	.407
*CDK4* amplification	16 (9.7%)	11 (33.3%)	5 (13.2%)	.051

Variables	All, *n* = 71	Group III, *n* = 33	Non-Ctx (Group II, IV), *n* = 38	*P*-value
*EGFR* alteration	18 (25.4%)	13 (39.4%)	5 (13.2%)	.015*
*PDGFRA* alteration	16 (22.5%)	6 (18.2%)	10 (26.3%)	.570
*PTEN* loss	35 (49.2%)	22 (66.7%)	13 (34.2%)	.009^**^

Abbreviations: OS, overall survival; SVZ, subventricular zone.

*P <.05, **P <.01, ***P <.001.

### Clinical Features and Genetic Alternations in Multiple GBM

We identified solitary, multifocal, and multicentric GBM lesions in 87 (70%), 28 (23%), and 9 (7%) patients, respectively. First, we compared solitary and multiple lesions; the latter were a combination of multifocal and multicenter lesions. Age, sex, preoperative KPS score, and Ki-67 labeling index were not significantly different between patients with solitary and multiple GBMs. *PDGFRA* alteration (*P* = .036) and *RB1* wild type (*P* = .031) were more common in multiple GBMs than in solitary GBM. *TERT*p mutation was more common in multiple GBMs than in solitary GBM; however, the difference was not statistically significant (*P* = .07; Table 4).

**Table 4.** Clinical and Genetic Differences Between Solitary and Multiple Glioblastoma Lesions

Variables	All (*n* = 124)	Solitary (*n* = 87)	Multiple (*n* = 37)	*P*-value
Mean age	66.4 ± 13.3	65.9 ± 14.5	67.8 ± 9.7	.459
*EGFR* alteration	35 (28.2%)	25 (28.7%)	10 (27.0%)	1.000
*PDGFRA* alteration	28 (30.6%)	15 (25.3%)	13 (43.2%)	.036^*^
*RB1* alteration	52 (41.9%)	42 (48.3%)	10 (27.0%)	.031^*^
*PTEN* alteration	79 (63.7%)	57 (65.5%)	22 (59.5%)	.546
*CDK4* amplification	23 (18.5%)	15 (17.2%)	8 (21.6%)	.617
*CDK6* amplification	3 (2.4%)	3 (3.4%)	0	.554
*TERT*p mutation	79 (63.7%)	60 (76.0%)	19 (51.4%)	.070
*TP53* alteration	60 (48.4%)	43 (49.4%)	17 (46.0%)	.845
*MGMT*p methylation	84 (67.7%)	59 (67.8%)	25 (67.6%)	1.000
Median OS (days)	618	688	488	.239

Abbreviation: OS, overall survival.

*P <.05, **P <.01, ***P <.001.

### OS Differences in Imaging Findings

**Figure 2.**
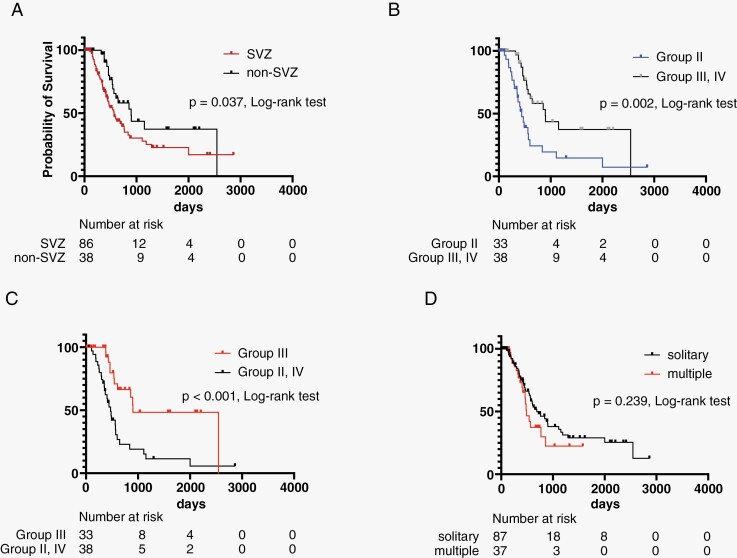
Differences in overall survival (OS) in imaging findings. (A) The subventricular zone (SVZ) group had a significantly worse OS than the non-SVZ group. (B) Group II had a significantly worse OS than the other groups (groups III and IV). (C) Group III had a better prognosis than the other groups (groups II and IV). (D) A comparison between solitary and multiple GBMs showed no significant difference in OS.

The SVZ group (CEL groups I and II) had a significantly worse OS than the non-SVZ group (median survival: 567 vs. 897 days, *P* = .037; Figure 2A). Among the 4 groups, group II had a poor prognosis than the other groups (groups I, III, and IV) (441 vs. 768 days, *P* = .004), and group III had a more favorable prognosis than the other groups (groups I, II, and IV) (901 vs. 566 days, *P* = .008; Supplementary Figure 2A and B]. Subsequently, group I was excluded considering its heterogeneous characteristics. CEL group II had a significantly worse OS (441 vs. 897 days, *P* = .002) and group III had a better prognosis (901 vs. 473 days, *P* < .001) than the other groups (Figures 2B and C). A comparison between solitary and multiple GBMs showed no significant difference in OS (688 vs. 488 days, *P* = .239; Figure 2D).

## Discussion

### Location of CELs and Gene Alterations

GBM is extremely heterogeneous and highly treatment resistant. It has the worst prognosis among central nervous system tumors. The possibility that tumor localization affects the prognosis of patients with GBM has been discussed. Previous reports have indicated that periventricular GBM and ventricular entry during surgery are associated with distant metastasis and intrathecal dissemination,^24,25^ and there was a rebuttal to this conclusion.^26^ The association of SVZ–GBM with high invasiveness and multiple lesions was first reported in 2007, and SVZ–GBM has since been associated with poor progression-free survival and OS.^6,9,27^ RTKs are commonly altered in GBM, with *EGFR* mutations and amplifications being the most frequent RTK alterations, occurring in approximately 45% of GBM tumors.^1^*EGFR* induces the proliferation of NSCs in the SVZ and inhibits cell differentiation.^28–30^ The inhibition of PI3K/Akt signaling downstream of RTK has also been reported to inhibit glioma growth in the SVZ,^31^ suggesting that RTK is an important therapeutic target for GBM tumorigenesis. A previous study showed no difference in *EGFR* amplification or *PTEN* loss between SVZ and non-SVZ GBMs in 26 newly diagnosed *IDH* wild-type GBM cases.^32^ This study showed that the frequency of *EGFR* amplification in *IDH* wild-type GBM was 46% (12/26) and that of *PTEN* loss was 69% (18/26). Our study included 24% (30/124) of *EGFR* amplification and 50% (62/124) of *PTEN* loss cases. The frequency of *EGFR* amplification in Japanese patients has been reported to be lower than that in other patients, which is consistent with the results of the present study.^33,34^ The present study included a larger cohort than did previous reports and showed an association between *EGFR* and *PTEN* status and cortical lesions.

Using our NGS panel, we showed that several driver genes were associated with the localization and multiplicity of CELs in GBM. The present study differs from previous reports because in addition to comparing the SVZ and non-SVZ groups, CEL group I was excluded, and cases clearly localized to the cortex or SVZ were compared. In addition to *EGFR* amplification, mutations were also included to demonstrate their relationship with tumor localization. Our study suggests for the first time that *PDGFRA* alteration is associated with multiple lesions. There was no significant relationship between *PDGFRA* alteration and CEL localization; however, the relationship between PDGFRA alteration and poor removal and survival rates was clinically meaningful.

*EGFR* alterations are more common in cortical lesions with a good removal rate, suggesting that this is one of the reasons why *EGFR* alteration has a good prognosis. Alternatively, the Ki-67 level was clearly lower in the *EGFR* alteration group, and there may be a significant difference in surgical outcomes and tumor aggressiveness. In addition to *EGFR* alteration, *PTEN* loss was significantly associated with cortical lesions. In a mouse model of SVZ NSCs transfected with mutated *p53*, *PTEN*, and *EGFR*, mutant cells migrated from the SVZ to remote areas of the brain and formed high-grade gliomas.^35^ To the best of our knowledge, this is the first radiogenomic study to show consistent results in humans. *EGFR* has been shown to affect the ability of cells to migrate remotely from the SVZ, which explains the high incidence of *EGFR* alteration in group III.

The origin of GBM is assumed to be the NSCs/neural progenitor cells (NPCs) and oligodendrocyte progenitor cells (OPCs) of the SVZ,^36–39^ and GBM is presumed to originate from cells years before diagnosis.^40^ NSCs in the SVZ have a genetically distinct feature from other localizations that may influence the development and progression of GBM.^11,12^ NSCs have the ability to differentiate into neurons and glial cells and are abundant in fetal tissues. Owing to their high proliferative potential, NSCs have been implicated in the development of GBM. Neftel et al. focused on the different states of tumor cells in GBM and found that 4 cell types exist: NPC-like, OPC-like, astrocyte-like, and mesenchymal-like cell morphologies.^41^ The marker of NPC-like GBM is *CDK4* amplification. In our study, *CDK4* amplification was more common in periventricular lesions (group II), which may reflect the characteristics of NPC-like GBM and tumor localization.

RTK status affects the proliferative potential.^42,43^ We showed that *EGFR* and *PDGFRA* alterations had contrasting characteristics regarding Ki-67 scores. Ki-67 is a known predictor of proliferative potential in various cancer types; however, its expression is heterogeneous, and the details of its association with specific genetic mutations in GBM remain unclear. *PDGFRA* alteration is a marker of OPC-like cells, which are less differentiated and may indicate high proliferative potential.

### Multiple CELs and Gene Alterations

Multiple GBM lesions are associated with secondary malignancies, a family history of cancer, germline p53 mutations, and a poor prognosis.^17,44,45^ There have been several reports on the genetic differences between solitary GBM and multiple GBM; however, these are limited to several major genes, and only a few studies have used comprehensive gene panels. A previous report showed that the frequency of *EGFR* mutation and *EGFR/PTEN* concurrent mutation is higher in multiple GBM and that multicentric GBM has fewer *CDK4* mutations and more *CDKN2A/B* mutations than multifocal GBM.^19^ Furthermore, a previous study showed that multifocal GBM arises from *PTEN* loss or *TERT*p mutations and develops through the RTK/PI3K, p53, and RB1 pathways.^18^ Other reports have shown that *TERT*p mutation is strongly correlated with multiple GBM lesions.^34^ In our study, *TERT*p mutation was more common in the multiple GBM group than in the solitary GBM group, although the difference was not statistically significant. In addition, our results showed that multiple GBMs were strongly correlated with *RB1* wild type, but we have not found similar reports for other cancers. A recent report indicated that *RB1* alterations affect the prognosis of GBM^46^; however, there are no studies on multiple lesions, and future research is needed.

The present study has some limitations. First, it was a retrospective study that is susceptible to selection bias. Second, because GBM is a histologically and genetically heterogeneous tumor, the analysis of some specimens removed from multifocal and multicentric GBM may not reflect the entire tumor. Additionally, in this study, bulk sequencing was performed on the portion of the tumor mass that was sampled, and the cell density was secured. Detailed data on where the collected tumor corresponds to on MRI have not been recorded. In the future, it is desirable to use neuronavigation to record the location of samples taken intraoperatively, create a database, and conduct prospective studies. Third, our cohort had a small number of patients in certain groups, such as CEL group IV and multicentric gliomas. Therefore, the clinical characteristics of each group and detailed subgroup analyses were unavailable. More cases are needed for future studies. Finally, this study was conducted only in Japanese patients. Recent reports suggest that the frequency of *EGFR* amplification in Asian cohorts is lower than that in Western cohorts,^47,48^ and genetic differences between races are controversial.

In conclusion, *EGFR* alteration was associated with cortical lesions, favorable removal rates, and low Ki-67 scores. In contrast, *PDGFRA* alteration correlated with high proliferative potential and multiple lesions. These results might have influenced the differences in prognosis among alterations in the RTKs. We considered *EGFR* and *PDGFRA* status as important markers of different localizations and clinical courses in GBM. Our results suggest that clarifying the association between driver genes and tumor localization may be useful in clinical practice, including prognosis prediction.

## Supplementary Material

vdad110_suppl_Supplementary_Material

## Data Availability

All data used and analyzed in this study are available from the corresponding author on reasonable request.
