# Supplementary material for: Alterations in EGFR and PDGFRA are associated with the localization of contrast-enhancing lesions in glioblastoma
Source: Neurooncol Adv. 2023 Sep 2;5(1):vdad110. doi: 10.1093/noajnl/vdad110 (PMC10516461; doi:10.1093/noajnl/vdad110)
Supplement: vdad110_suppl_Supplementary_Material [file vdad110_suppl_supplementary_material.zip › vdad110_suppl_Supplementary_Figures_1.pptx]

## Slide 1
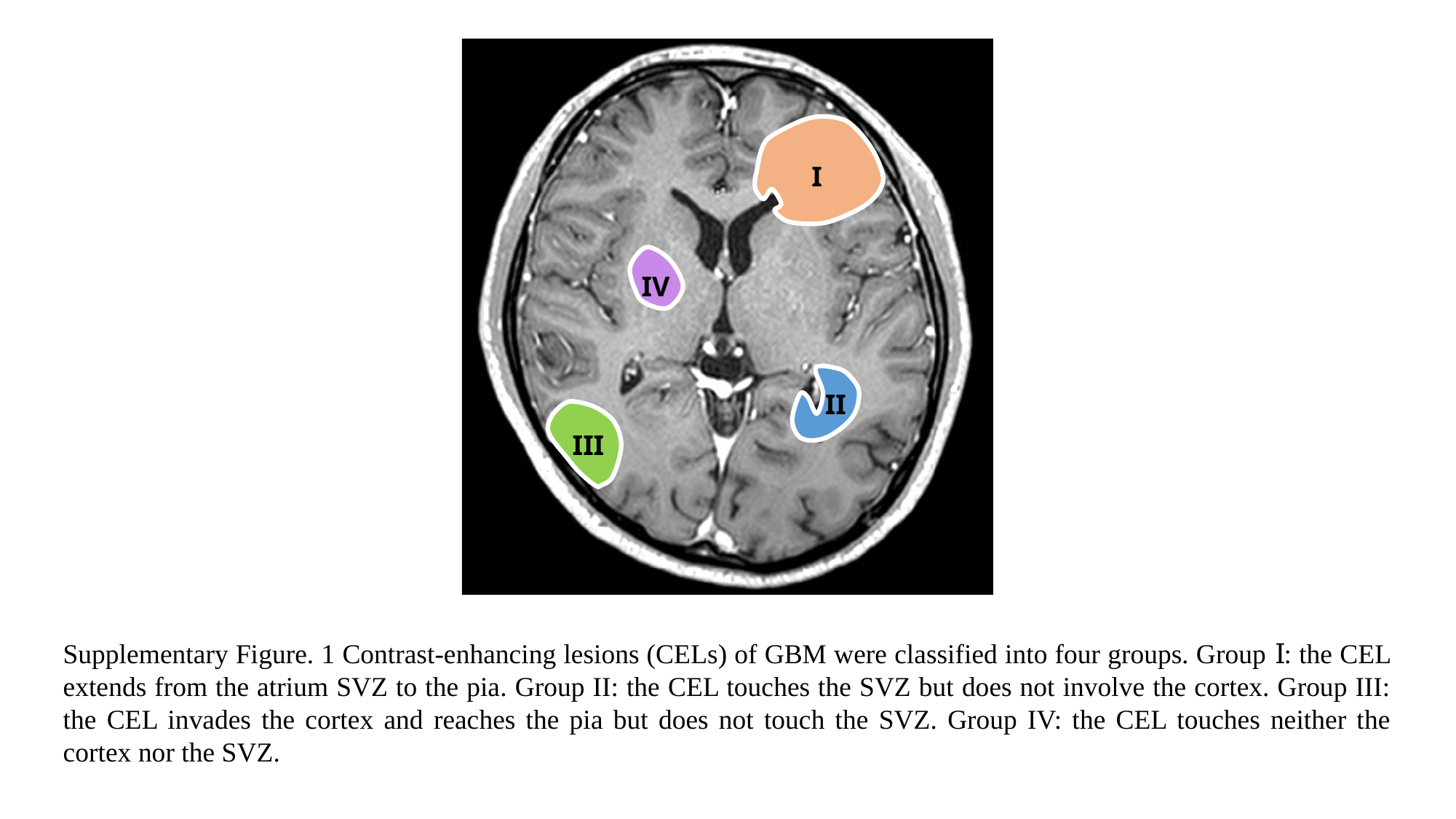

I
IV
II
III
Supplementary Figure. 1 Contrast-enhancing lesions (CELs) of GBM were classified into four groups. Group Ⅰ: the CEL extends from the atrium SVZ to the pia. Group II: the CEL touches the SVZ but does not involve the cortex. Group III: the CEL invades the cortex and reaches the pia but does not touch the SVZ. Group IV: the CEL touches neither the cortex nor the SVZ.
