# Supplementary material for: Alterations in EGFR and PDGFRA are associated with the localization of contrast-enhancing lesions in glioblastoma
Source: Neurooncol Adv. 2023 Sep 2;5(1):vdad110. doi: 10.1093/noajnl/vdad110 (PMC10516461; doi:10.1093/noajnl/vdad110)
Supplement: vdad110_suppl_Supplementary_Material [file vdad110_suppl_supplementary_material.zip › vdad110_suppl_Supplementary_Figures_2.pptx]

## Slide 1
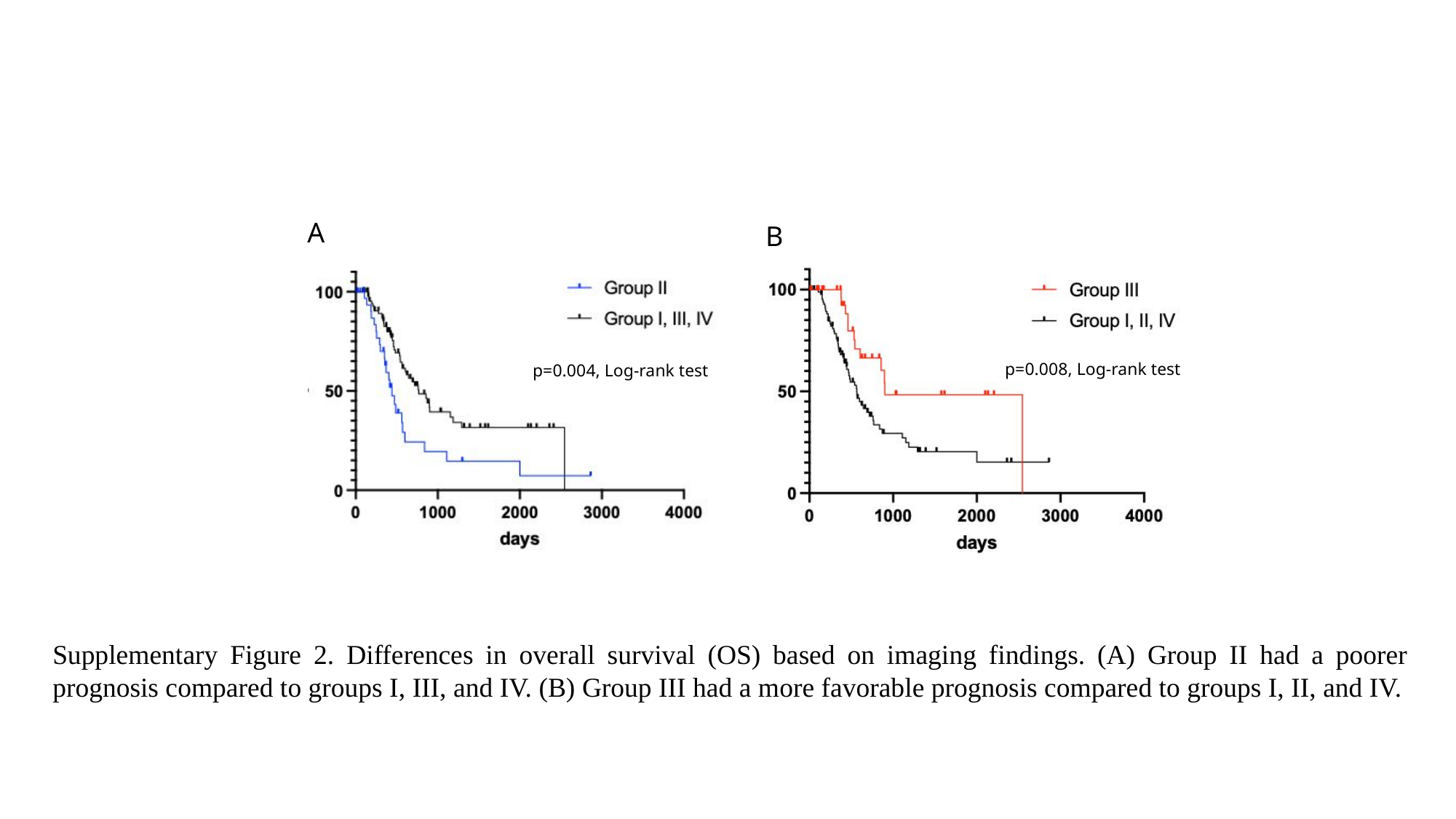

A
B
p=0.008, Log-rank test
p=0.004, Log-rank test
Supplementary Figure 2. Differences in overall survival (OS) based on imaging findings. (A) Group II had a poorer prognosis compared to groups I, III, and IV. (B) Group III had a more favorable prognosis compared to groups I, II, and IV.
